# Training Positive Rumination in Expressive Writing to Enhance Psychological Adjustment and Working Memory Updating for Maladaptive Ruminators

**DOI:** 10.3389/fpsyg.2020.00789

**Published:** 2020-05-13

**Authors:** Hongfei Yang, Huizhong Li

**Affiliations:** Department of Psychology and Behavioral Sciences, Zhejiang University, Hangzhou, China

**Keywords:** positive rumination training, expressive writing, maladaptive ruminators, psychological adjustment, working memory updating

## Abstract

Rumination is associated with psychological adjustment and working memory (WM) capacity. Studies have shown that psychological interventions can reduce negative rumination and improve psychological adjustment and WM capacity. The present study investigated the effect of positive rumination training in expressive writing on psychological adjustment and WM updating capacity. Within an experimental design, positive rumination was manipulated for 10 participants who were maladaptive ruminators in an experiment using a 5-week training compared to the control group with nine participants. Results revealed significant enhancement of psychological adjustment and the response time (RT) of WM updating in the experimental group but not in the control group. The two groups did not show significant difference of all the variables in pretest. However, the experimental group showed significantly better outcomes than the control group in posttest. The results suggest that positive rumination training in expressive writing is effective and rumination has a causal influence on WM updating capacity.

## Introduction

### Positive and Negative Rumination

Rumination was originally defined as repetitive and passive thinking about negative affects and their possible causes and consequences (Nolen-Hoeksema, 1991). It is perceived as a risk factor for psychopathology, especially for depression (Papageorgiou and Siegle, 2003). Consequently, the majority of previous studies on rumination focused on its pathological aspects. In these studies, individuals with high and low levels of negative rumination were usually identified as ruminators and non-ruminators, respectively (e.g., Davis and Nolen-Hoeksema, 2000; Beckwé and Deroost, 2016).

However, some researchers have pointed out that rumination can be beneficial. For example, Martin and Tesser (1996) proposed that positive forms of rumination help individuals find alternative solutions to their problems. Watkins (2008, 2016) proposed that unconstructive repetitive thinking or abstract analytic rumination focusing on the causes, consequences, and meaning of a problem is maladaptive, whereas constructive repetitive thinking or concrete-experiential rumination focusing on a plan, a decision, and some kind of action to deal with it is adaptive. Similarly, Cann et al. (2011) proposed that intrusive rumination involving unsolicited invasions of thoughts about a negative event is maladaptive, whereas deliberate rumination involving voluntary focus on understanding events and their implications is adaptive. Importantly, Feldman et al. (2008) insisted that response to positive affect is equally important for emotion regulation. In his Response to Positive Affect Scale (RPAS), Emotion-focused and Self-focused Positive Rumination are adaptive whereas Dampening is maladaptive. Of note, while the Rumination Response Scale (RRS, Treynor et al., 2003) with reflection and brooding subscales has been widely used to assess maladaptive rumination, some researchers found that reflection is adaptive while brooding is maladaptive (Whiteman and Mangels, 2016).

Considering that previous studies have tapped adaptive or positive and maladaptive or negative rumination on either negative or positive affect but not both, we tried to integrate them by defining rumination as repetitive thoughts about both positive and negative affect and they function either positively or negatively regarding psychological adjustment. We then proposed a 2 (positive and negative affect) × 2 (positive and negative rumination) model of rumination and developed the Positive and Negative Rumination Scale (PANRS) (Yang et al., 2018). In the same study, we also identified three types of ruminators by conducting a two-step cluster analysis using Positive and Negative Rumination subscale scores as variables: adaptive ruminators (i.e., individuals with high positive rumination and low negative rumination), maladaptive ruminators (i.e., individuals with low positive rumination and high negative rumination), and non-ruminators (individuals with low positive rumination and low negative rumination). The adaptive ruminators have the best psychological adjustment followed by non-ruminators and maladaptive ruminators successively. Accordingly, maladaptive ruminators are more vulnerable to mental disorder than the other two types of ruminators and need preventive intervention.

From the perspective of positive psychology, the absence of well-being – positive emotion, engagement, purpose, positive relationships, positive accomplishment – is an important risk factor of mental disorder and bringing about well-being may be one of the best weapons against mental disorder (Seligman, 2008). Therefore, promoting positive rumination would be likely to help reduce negative rumination and enhance psychological adjustment. Consequently, we developed the positive rumination training method and applied it in psychological counseling and therapy (Xuan et al., 2019; Yang, 2019; Yang and Yang, 2019). Importantly, in line with the notion that rumination is a habitual behavior (Martell et al., 2001; Chang et al., 2017), both positive and negative rumination can be viewed as habitual thinking styles developed in daily life that can be enhanced or reduced through intentional training.

### Expressive Writing

Expressive writing refers to the procedure of writing about emotionally upsetting experiences without paying attention to grammar or spelling (Pennebaker and Beall, 1986). Previous studies have shown inconsistent findings regarding its effect on psychological adjustment. For example, in a systematic review, Riddle et al. (2016) found a significant effect of expressive writing on reducing trauma and improving general psychological health for caregivers but no effect on changing their depression, anxiety, physical symptoms, quality of life, or burden. Relatedly, in a systematic review, Lee et al. (2016) found that expressive writing improved the quality of life for women with breast cancer, but did not reduce their negative emotions. They then pointed out the “importance of modifying the traditional expressive writing protocol to enhance its efficacy” (p. 99). Furthermore, in a recent meta-analysis, Reinhold et al. (2018) found no significant long-term effects of expressive writing on depressive symptoms. However, the higher number of sessions and the more specific writing topic produced larger effects. Therefore, they proposed that brief, self-directed expressive writing should not be considered as an effective method to decrease depressive symptoms in physically healthy adults and that future research should employ longer and counselor-directed expressive writing.

Regarding the effects of expressive writing on rumination, Gortner et al. (2006) found that writing for 20 min on 3 consecutive days significantly reduced depression level 6 months later. This effect was mediated by changes of brooding. Relatedly, Sloan et al. (2008) found that the same number of sessions significantly reduced depression 2, 4, and 6 months later. Brooding ruminative style moderated the effects of expressive writing such that participants with higher brooding scores reported significantly lower depression level relative to those with lower brooding scores. Recently, Glass et al. (2019) found that a modified framework of expressive writing significantly reduced rumination for adults who had a trauma, or significant emotional/physical upheaval, within the past year.

These findings suggest that expressive writing could be modified to reduce negative rumination such as brooding.

### Working Memory Updating

Working memory (WM) updating is a core component of WM and refers to one’s ability to monitor and dynamically manipulate WM contents, to evaluate its relevance for the current task, and to replace it with newer, more relevant information (see Zuber et al., 2018). Theoretically, when individuals have deficits in WM updating, they could only process old information in WM because they are unable to update it. If they repeatedly process such old negative information in negative ways, they are likely to become negative ruminators (Chang et al., 2017). On the other hand, if individuals habitually process old negative information in negative ways, they would not update the information. Thus, they are likely to have WM deficits (van Vugt et al., 2018; Vrshek-Schallhorn et al., 2019). Empirically, there is evidence to show the association between negative rumination and deficits in WM updating. For example, Bernblum and Mor (2010) found that, compared with non-brooders, brooders showed slowed refreshing (of emotional and neutral words) when relevant emotional words were presented. Similarly, Quinn and Joormann (2015) found that WM updating performance did not show difference between brooders and non-brooders before stress induction. After stress induction, brooders showed significantly poorer WM updating performance than non-brooders. Similarly, Kaiser et al. (2015) found that individuals with a high RRS score were slower to update WM after exposure to criticism from family members or partners. Recently, Vrshek-Schallhorn et al. (2019) found that rumination predicted greater difficulty in WM updating under the condition without induced stress. These findings suggest that there is a causal relationship between rumination and WM updating though if this relationship is one-directional or bidirectional remains unclear.

Of note, previous studies have found that psychological interventions can improve WM capacity. For example, expressive writing (Yogo and Fujihara, 2008), mindfulness training (Mrazek et al., 2013), problem solving training (Swanson, 2015), and social regulation of emotion (Flores and Berenbaum, 2017) can all improve WM capacity. These findings implicate that psychological interventions have a causal influence on WM.

### The Present Study

Overall, previous findings indicate that positive and negative rumination are associated with psychological adjustment and some psychological interventions such as positive rumination training and expressive writing can reduce negative rumination and improve psychological adjustment. The previous findings also indicate that negative rumination was associated with WM updating deficit and some psychological interventions such as expressive writing can improve WM capacity. However, the association of positive rumination with WM updating and if positive rumination training can improve WM updating remains unknown. Consequently, the present study aimed to combine positive rumination training with expressive writing using a counselor-directed protocol, and examine its effect on psychological adjustment and WM updating for maladaptive ruminators. Following the dual-factor model of mental health (Greenspoon and Saklofske, 2001; Suldo and Shaffer, 2008), life satisfaction and depression were used as positive and negative indicators of psychological adjustment in the present study. We assume that this training can improve both psychological adjustment and WM updating by increasing positive rumination and reducing negative rumination.

## Materials and Methods

### Participants

Seventy undergraduates were recruited from the authors’ university. Using cluster analysis, 22 maladaptive ruminators were identified, whose scores of negative rumination were 2.30 or higher. Among them, 20 participants were randomly selected and assigned to either the experimental or control groups, each with 10 participants. The experimental group had three males and seven females with a mean age of 18.60 (SD = 0.84, range = 18–20). One participant in the control group did not finish the posttest, leaving a final sample of 9 (two males and seven females). The mean age was 19.98 (SD = 1.20, range = 18–21). Students were recruited after class and volunteered to participate in the study without compensation. They had no mental disorders.

### Design and Procedure

A 2 (group: experimental and control group) × 2 (test: pre- and post-test) design was employed. Participants first consented to take part in the study. They then completed the pretest and were randomly assigned to experimental or control group. The experimental group received 5-week intervention with 30 min each session and one session per week. The control group was in the waiting list without intervention. When the experiment was finished, the control group participants received the same intervention. In both pre- and post-test, participants completed a brief demographic questionnaire followed by measures and, finally, the 2-back emotion updating.

### Materials

#### Measures

Rumination was measured by the PANRS (Yang et al., 2018), which is a 23-item scale with two second-order factors: Positive and Negative Rumination. Positive Rumination consists of two first-order factors, i.e., Enjoy Happiness (six items, e.g., “Think ‘How wonderful life is”’) and Positive Coping (four items, e.g., “Think ‘What I can do for it”’). Negative Rumination consists of three first-order factors, i.e., Suppress Happiness (five items, e.g., “Think ‘Fall from the pinnacle of one’s power”’), Self-Deny (three items, e.g., “Think ‘I am a useless person”’), and Negative Attribution (five items, e.g., “Think ‘Misfortunes never come singly”’). Items were rated on a four-point Likert scale ranging from 1 (*not at all*) to 4 (*always*). Higher scores on the positive and negative subscales of PANRS indicate greater positive and negative rumination, respectively. The five first-order factors and the two second-order factors were found to have satisfactory internal consistency (Cronbach’s α = 0.71–0.85).

Life satisfaction and depression were used as positive and negative indicators of psychological adjustment. Life satisfaction was measured by the Satisfaction With Life Scale (SWLS; Diener et al., 1985). It has five items (e.g., “I am satisfied with my life”) rated on a seven-point Likert-type scale ranging from 1 (strongly disagree) to 7 (strongly agree). Higher scores on the SWLS indicate greater life satisfaction. The scale was found to have satisfactory internal consistency (Cronbach’s α = 0.87).

Depression was measured by the depression subscale of Symptom Checklist-14 (SCL-14, see Prinz et al., 2013). It has six items (e.g., feeling lonely) rated on a five-point Likert scale ranging from 0 (never) to 4 (very often). Higher scores on the depression subscale indicate greater depression. The scale was found to have satisfactory internal consistency (Cronbach’s α = 0.87).

In the present study, average scores were used for all the above measures.

**FIGURE 1:**
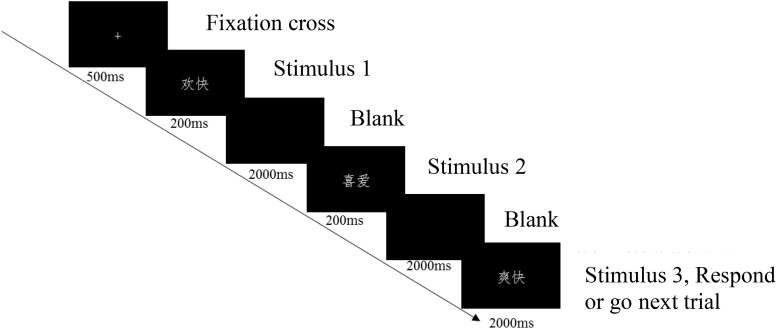
Illustration of the 2-back task.

Working memory updating capacity was measured with a 2-back emotion updating task that is illustrated in Figure 1. As the figure shows, a word was presented for 200 ms in each trial. Participants were instructed to respond as quickly and accurately as possible regarding whether Stimulus 3 matched Stimulus 1, by pressing the “F” or “J” button on the computer keyboard with their left or right index finger. The assignment of keys to “yes” and “no” answers was counterbalanced across participants.

The stimuli were 72 two-character words selected from the Chinese Affective Words System (CAWS, Wang et al., 2008), with each stimulus category (positive, neutral and negative) containing 24 words. Every word consists of two Chinese characters. Examples of positive, neutral, and negative adjectives are “Man-yi (??, satisfactory),” “Fa-lv (??, law),” and “Ai-shang (??, grief),” respectively. The CAWS provides valence and arousal norms that were developed based on a nine-point scale ranging from 1 (*most negative/arousing*) to 9 (*most positive/arousing*), respectively, where 5 is neutral for both. The three categories of words differed significantly in valence (positive = 6.85 ± 0.23, neutral = 5.20 ± 0.23, negative = 3.06 ± 0.24; *F*(2,69) = 1597.75, *p* < 0.001, pairwise comparisons: *p* < 0.001). The arousal of positive (5.04 ± 0.70) and negative (4.86 ± 0.35) words was significantly higher than that of neutral words (3.94 ± 0.53) [*F*(2,69) = 27.52, *p* < 0.001], whereas the difference between the arousal of negative and positive words was not significant (*p* = 0.252).

Prior to the formal experiment, participants performed at least 15 practice trials for each memory load to get familiar with the task. The stimuli in practice trials were neutral faces that were not used in the formal experiment. The practice phase was stopped when participants achieved 80% correct responses.

The 2-back task of formal experiment consisted of three blocks (positive, neutral, and negative blocks; see also Han et al., 2013; Zhang et al., 2018). In each block, there were 24 trials, with half “match” trials and the other half “non-match” trials. Stimuli in the same block were randomly selected for any trials. “Match” and “non-match” trials were also presented randomly. The block order was counterbalanced across participants. Participants took 1-min break between blocks.

#### Intervention

Participants in the experimental group received positive rumination training in expressive writing. This novel approach expands the Pennebaker Paradigm into a positive counseling framework that involves positive rumination and counselor-oriented protocol. It recognizes that while a simple expressive writing sequence may be effective, participants would benefit more from a positive writing style. Specifically, participants were instructed to write about a very upsetting event they experienced and their “deepest emotions and thoughts” about it. To preserve the unimpeded free-association process of the original writing paradigm, participants could choose the same or a different event for subsequent writing sessions. All the instructions for five sessions were presented in Table 1. The experimenter knocked on the laboratory door (without entering) once the 30-min writing period had ended to cue the participant to stop writing.

**TABLE 1:** Intervention plan.

**Session**	**Topic**	**Instruction**
1	Positive rumination on negative events and emotions	Please recall the most miserable, frustrated, or difficult life events you experienced and your deep emotions and thoughts about them. Then, please think about their positive effect on you and write it down. You may like to follow these guidelines: (1) How did you cope with the upsetting events and what good consequences your coping lead to you. (2) After the events, how did you live your life and made it better and better? (3) When your negative emotions such as sadness went away, did the events bring you some unexpected achievements? For example, perhaps you became aware of your personal strengths that you did not realize you had, perhaps you knew better about your life goals or what you really wanted than ever before. Life events have two sides. Please face them realistically and objectively. When you realize their hurts to you, please keep aware of their positive effects on you and prevent yourself from brooding in sadness.
2	Positive rumination on negative events and emotions	You may write the same topic as in Session 1 or write a new topic. (Other introduction is the same as in Session 1.)
3	Positive rumination on positive events and emotions	Please recall the best life events or the happiest time you experienced. Choose one or a couple of them and imagine as if you are in that time. Focus on your feelings, ideas, and emotions and write them down.
4	Positive rumination on positive events and emotions	You may write the same topic as in Session 1 or write a new topic. (Other introduction is same as in Session 3.)
5	Strengthening positive rumination	In the past four sessions, you recalled those miserable, frustrated, and difficult events and tried to think about the positive effects they led to you. Meanwhile, you also recalled those happiest life events and their deep positive effects on you. In this last session, you may like to choose one of the (miserable or happy) events you have written before or a new event and write down your feelings and thoughts with the same rumination style.

The experimental group was required to finish a homework, i.e., to write one happy event and one unhappy event every day and thought about them three times using any related three items of the positive rumination subscale as guidelines (see Table 2). From the second session on, the experimenter gave feedback to participants about their last expressive writings and provided some guidance. For example, two participants only wrote down the events without writing about their feelings and thoughts. After receiving comments and suggestions, they made adjustments in the following sessions. The experimenter also paid attention to psychological issues expressed in writings and provided help to them. Afterward, the experimenter randomly chose a positive or negative event in their homework and discussed with them how to think about it in the guidance of positive rumination items.

**TABLE 2:** Items of positive rumination subscales.

**Enjoy happiness**	**Positive coping**
1. Think “How wonderful life is.”	1. Think to calm down.
2. Think “I am proud of myself.”	2. Think “What I can do for it.”
3. Think “I am energetic.”	3. Think “A fall into the pit, a gain in your wit.”
4. Think “How happy I am.”	4. Think to cheer up.
5. Think “I am great.”	
6. Think “I have a bright future.”	

The experimenter is the first author who was a graduate student majoring in counseling psychology and achieved a certificate of third class national psychological counselors. The second author is a graduate program supervisor in counseling psychology and has a certificate of national psychological counselor trainers.

### Data Analysis

Following the method widely used to identify outliers of data (e.g., Baddeley, 2003; Bernblum and Mor, 2010; Berger et al., 2018), response times (RTs) faster than 200 ms or slower than 1200 ms or 2.5 SD above or below the respective group’s *M* were excluded. Single trials with less than 80% accurate responses were also excluded. Then, paired-sample *t* tests from pretest through posttest were used to determine the intervention effectiveness. Cohen’s *d* effect sizes were calculated with 0.20–0.49, 0.50–0.79, and 0.8 or higher representing small, medium, and large effect sizes, respectively (Cohen, 1988). Posttest outcome was assessed with analysis of covariance (ANCOVA) of post-treatment scores controlling for pretest scores. This procedure is more statistically powerful and parsimonious than a repeated-measures multivariate analysis of variance (MANOVA; see Bond et al., 2008). Partial eta squared (η^2^) effect sizes were calculated, with 0.01, 0.06, and 0.14 representing small, medium, and large effect sizes, respectively (Cohen, 1988). Statistics were performed by using SPSS 20.0 for Windows (SPSS Inc., Chicago, IL, United States).

## Results

**FIGURE 2:**
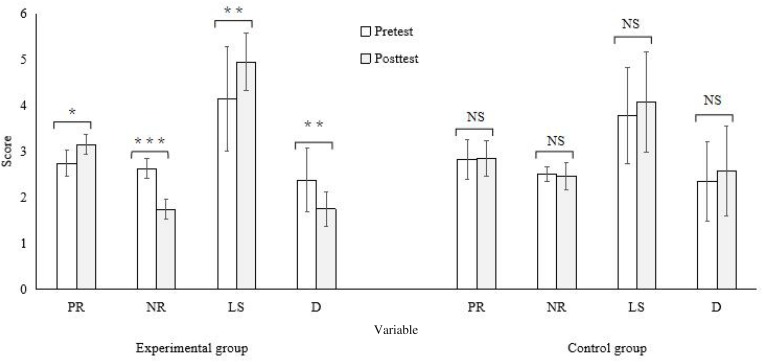
Comparisons between pre- and post-test scale scores for two groups (*P < 0.05, **P < 0.01, ***P < 0.001, NS, not significant; PR, positive rumination; NR, negative rumination; LS, life satisfaction; D, depression).

**FIGURE 3:**
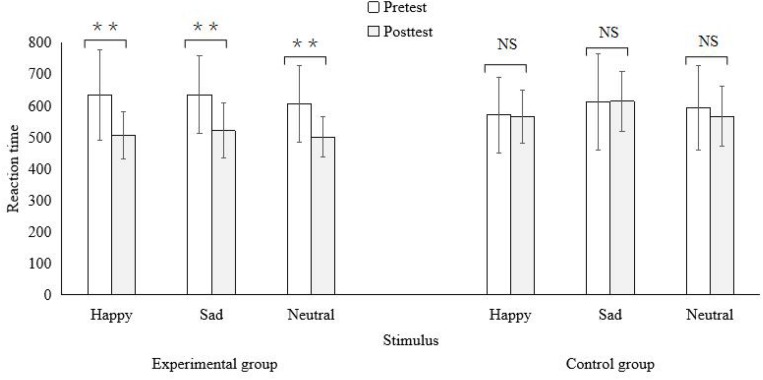
Comparisons between pre- and post-test reaction time for two groups (**P < 0.01, NS, not significant).

All variable scores were presented in Table 3. Independent-samples *t* tests revealed no significant group differences in all pretest scores (*t* = −1.32 to −1.55, *p*s > 0.05, *d* = −0.67/0.70). As shown in Table 3 and Figures 2, 3, the scores of positive rumination and life satisfaction increased significantly (*t* = −3.19/−3.10, *p*s < 0.05, *d* = −1.62/−0.87) while the scores of negative rumination, depression, and the RTs to all three types of words decreased significantly (*t* = 3.32–8.86, *p*s < 0.01–0.001, *d* = 1.07/4.42) after a 5-week intervention for the experimental group. The effect sizes were large. In contrast, all the scores did not change significantly across the 5 weeks for the control group. Moreover, the ACC (accuracy) did not show significant changes for both groups. ANCOVA showed significant differences in the posttest scores of all variables (*F* = 4.33–33.34, *p*s < 0.10–0.001, η^2^ = 0.21–0.68) except for ACCs between two groups when the pretest scores were controlled for. The experimental group scored significantly higher on positive rumination and life satisfaction, and lower on negative rumination and depression than the control group. The experimental group’s RTs were significantly shorter than the control group’s. The effect sizes were large.

**TABLE 3:** Mean (with SD) scores and comparisons.

**Variables**	**Experimental group (*n* = 10)**				**Control group (*n* = 9)**				**ANCOVA**	
	
	**Pretest**	**Posttest**	***t***	***d***	**Pretest**	**Posttest**	***t***	***d***	***F***	η^2^
Positive rumination	2.74 (0.29)	3.15 (0.21)	−3.19*	−1.62	2.83 (0.43)	2.84 (0.39)	−0.14	−0.02	5.97*	0.27
Negative rumination	2.63 (0.21)	1.74 (0.21)	8.86**	4.24	2.50 (0.16)	2.46 (0.29)	0.48	0.17	33.34***	0.68
Life satisfaction	4.14 (1.14)	4.94 (0.62)	−3.10*	–	3.78 (1.05)	4.07 (1.09)	−0.08	−0.27	4.33^†^	0.21
Depression	2.38 (0.69)	1.75 (0.37)	3.32**	1.15	2.35 (0.87)	2.57 (0.97)	−0.64	−0.24	6.95*	0.30
RT of WM updating										
Happy	633.21 (143.63)	505.84 (75.72)	3.64**	1.11	569.79 (119.06)	563.38 (84.00)	0.35	0.06	11.59**	0.42
Sad	634.08 (123.12)	519.57 (87.25)	3.92**	1.07	611.51 (152.93)	612.97 (93.87)	−0.06	−0.01	18.49**	0.54
Neutral	605.85 (122.10)	500.00 (63.64)	3.53**	1.09	592.91 (134.89)	566.17 (95.85)	1.57	0.07	11.26**	0.41
ACC of WM updating										
Happy	0.96 (0.05)	0.97 (0.03)	−0.55	−0.17	0.97 (0.04)	0.97 (0.04)	−0.23	0.00	0.08	0.01
Sad	0.95 (0.04)	0.97 (0.04)	−1.18	−0.50	0.97 (0.03)	0.94 (0.05)	0.96	0.01	0.58	0.04
Neutral	0.97 (0.03)	0.98 (0.03)	−1.02	−0.32	0.96 (0.04)	0.96 (0.03)	−1.08	0.00	0.00	0.00

^†^*p* < 0.10, *p < 0.05, **p < 0.01, ***p < 0.001.

## Discussion

The present study used positive rumination training in expressive writing to intervene in maladaptive rumination. The results provide evidence for its effectiveness. Specifically, the present study found that positive coping is a productive way of attending to negative emotions. This is consistent with previous findings that show that deliberate rumination (Shigemoto et al., 2017) and problem-solving (Gortner et al., 2006) are productive ways. Moreover, the present finding indicates that enjoying happiness is a productive way of attending to positive emotions. From the feedback of participants, enjoying happiness was a new and somewhat difficult thinking style to learn because they had habitually thought about the negative aspect of positive emotion and rarely thought about its positive aspect in daily life. After intervention, they realized that this thinking style is helpful to broaden their cognitive scope and improve their happiness. These findings are consistent with previous theoretical perspectives and empirical research results about the effects of positive and negative rumination on psychological adjustment (Martin and Tesser, 1996; Watkins, 2008, 2016; Cann et al., 2011; Yang et al., 2018). However, the relationship between rumination and some psychological adjustment indicators such as life satisfaction and depression might be bidirectional. For example, Elizabeth et al. (2018) found that the relationship between rumination and depression is reciprocal while Zanon et al. (2016) found that subjective well-being predicts rumination across time.

The present study also found that positive rumination training in expressive writing improved the WM updating capacity in terms of RT to all three types of words significantly, indicating that rumination has a causal influence on WM updating. This finding fits well with previous studies demonstrating that psychological interventions can improve WM capacity (Yogo and Fujihara, 2008; Mrazek et al., 2013; Swanson, 2015; Flores and Berenbaum, 2017). Specifically, the present finding indicates that positive rumination would facilitate WM updating, whereas negative rumination would impair it. This can be explained by the theoretical point that emotional regulation is a process of updating emotional information, i.e., to remove negative emotional information out of WM and replace it with a positive one (Levens and Gotlib, 2010, 2015). Thus, positive rumination might be understood as an emotional regulation strategy that helps individuals update emotional information quickly. In contrast, negative rumination causes individuals to update emotional information slowly.

This perspective also implies that the relationship between rumination and WM updating is most probably reciprocal and WM updating training would also improve positive rumination and reduce negative rumination. A recent study conducted by Peng et al. (2019) supports this point of view. They found that WM updating training can improve emotion regulation ability. However, some previous studies found that the effect of WM training did not transfer to rumination and depression (Onraedt and Koster, 2014; Wanmaker et al., 2015). A possible explanation is that the WM training was not intensive enough to produce effect transfer in their studies. Another possible explanation is that the effect transfers from positive rumination training to WM and from WM training to rumination are different regarding their underlying process. Of course, more research is needed to test these explanations.

It is worth noting that the present study did not find significant changes of ACC of WM updating across pre- and post-test for both groups, which is consistent with some previous studies (Zhao et al., 2013; Zhang et al., 2019). This might be due to the low difficulty of the task for our participants because the ACC ranged 94–97%.

### Limitations and Future Studies

The present study had a number of limitations. First, the sample size was small, which makes it difficult to generalize the findings. Future studies need to recruit more participants. Second, future studies need to measure more aspects of psychological adjustment such as reappraisal, resilience, anxiety, etc. Third, the present study only has one experimental group, making it difficult to clarify if the relationship between rumination and WM updating is reciprocal. Future studies would profit from having one more experimental group accepting WM updating training. Future studies would also profit from formal interviews with participants about the process of influence to deepen our understanding of their relationship. Finally, future studies need to examine if the mere act of writing has induced an effect on psychological adjustment and WM updating.

## Conclusion

Our findings suggest that positive rumination training in counselor-directed expressive writing is an effective method helping maladaptive ruminators to enhance psychological adjustment. This effect can transfer to the RT of WM updating. Future research is required to examine if the effect can transfer to the ACC of WM updating in more difficult tasks such as the 3-back task and if WM updating training can transfer its effect to positive and negative rumination and psychological adjustment. Future research is also required to examine if the present findings can be generalized to other samples such as students, individuals in workplace, and patients with depression.

## Data Availability Statement

The datasets generated for this study are available on request to the corresponding author.

## Ethics Statement

This study was carried out in accordance with the recommendations of the ethical standards of the Academic Research and Ethics Committee, Department of Psychology and Behavioral Sciences, Zhejiang University, China, with written informed consent from all subjects. All subjects gave written informed consent in accordance with the Declaration of Helsinki. The protocol was approved by the Academic Research and Ethics Committee, Department of Psychology and Behavioral Sciences, Zhejiang University, China.

## Author Contributions

HY is in charge of the research projects and the supervisor of HL who is a graduate student. HL finished the experiment and wrote the study in her thesis. HY finished the manuscript in English.

## Conflict of Interest

The authors declare that the research was conducted in the absence of any commercial or financial relationships that could be construed as a potential conflict of interest.
