# Corrigendum: Training Positive Rumination in Expressive Writing to Enhance Psychological Adjustment and Working Memory Updating for Maladaptive Ruminators

**DOI:** 10.3389/fpsyg.2020.01518

**Published:** 2020-06-26

**Authors:** Hongfei Yang, Huizhong Li

**Affiliations:** Department of Psychology and Behavioral Sciences, Zhejiang University, Hangzhou, China

**Keywords:** positive rumination training, expressive writing, maladaptive ruminators, psychological adjustment, working memory updating

In the original article, we neglected to include the funder Zhejiang Provincial Philosophy and Social Science Research Program of China, 17NDJC198YB and Humanities and Social Sciences of Ministry of Education Planning Fund of China, 17YJA190014 to author Hongfei Yang.

The following Funding section has been added to the article:

This research was supported by the Zhejiang Provincial Philosophy and Social Science Research Program of China, 17NDJC198YB and the Humanities and Social Sciences of Ministry of Education Planning Fund of China, 17YJA190014 (to HY).

The authors apologize for this error and state that this does not change the scientific conclusions of the article in any way. The original article has been updated.

